# Daily regulation of body temperature rhythm in the camel (*Camelus dromedarius*) exposed to experimental desert conditions

**DOI:** 10.14814/phy2.12151

**Published:** 2014-09-28

**Authors:** Hanan Bouâouda, Mohamed R. Achâaban, Mohammed Ouassat, Mohammed Oukassou, Mohamed Piro, Etienne Challet, Khalid El Allali, Paul Pévet

**Affiliations:** 1 Department of Neurobiology of Rhythms, CNRS UPR 3212, Institute for Cellular and Integrative Neurosciences, University of Strasbourg, Strasbourg, France; 2 Comparative Anatomy Unit (URAC CNRST 49), Hassan II Agronomy and Veterinary Institute, Rabat, Morocco; 3 Medecine and Surgical Unit of domestic animals, Hassan II Agronomy and Veterinary Institute, Rabat, Morocco

**Keywords:** Adaptive heterothermy, body temperature rhythm, camel, circadian clock, dehydration, food decrease, heat stress

## Abstract

In the present work, we have studied daily rhythmicity of body temperature (Tb) in Arabian camels challenged with daily heat, combined or not with dehydration. We confirm that Arabian camels use heterothermy to reduce heat gain coupled with evaporative heat loss during the day. Here, we also demonstrate that this mechanism is more complex than previously reported, because it is characterized by a daily alternation (probably of circadian origin) of two periods of poikilothermy and homeothermy. We also show that dehydration induced a decrease in food intake plays a role in this process. Together, these findings highlight that adaptive heterothermy in the Arabian camel varies across the diurnal light–dark cycle and is modulated by timing of daily heat and degrees of water restriction and associated reduction of food intake. The changed phase relationship between the light–dark cycle and the Tb rhythm observed during the dehydration process points to a possible mechanism of internal desynchronization during the process of adaptation to desert environment. During these experimental conditions mimicking the desert environment, it will be possible in the future to determine if induced high‐amplitude ambient temperature (Ta) rhythms are able to compete with the zeitgeber effect of the light–dark cycle.

## Abstract (precis)

e12151

In the Arabian camel, the “adaptative heterothermy” is characterized by a daily alteration (probably of circadian origin) of two periods. The findings highlight that adaptative heterothermy varies across the diurnal light–dark cycle and is modulated by timing of daily heat and degrees of water restriction and associated reduction of food intake.

## Introduction

Rhythmicity is a fundamental property of all living organism. In mammals, the circadian clock, located in the suprachiasmatic nuclei of the hypothalamus (SCN) is central for these rhythmic processes and plays a pivotal role to control numerous circadian biological rhythms such as those of melatonin synthesis and secretion, behavioral features, or body temperature (Tb). In most mammals, rhythmic Tb represents a robust output of the master clock in the SCN, widely used in fundamental and clinical research to determine properties of the SCN clock. In a recent work, we observed that the daily Tb rhythm in the camel is a true circadian rhythm entrainable by environmental cues (light–dark cycle as in other mammals, but also the ambient temperature (Ta) cycle). However, we also demonstrated that, in some specific experimental conditions, a direct effect of Ta on thermal regulation (thus on Tb) is present El Allali et al. (2013). This suggests that in camel, which is known to present specific responses to Ta, the daily Tb rhythm is controlled by a complex mechanism. The aim of the present work was thus to study the nature of daily Tb rhythm in the camel and to determine its relationship with the circadian system under challenging conditions mimicking the desert environment.

The Camel (*Camelus dromedaries*) is not only able to adapt its physiological processes to the hostile environment of arid and desert areas but also capable of maintaining synthesis of hair, wool, muscles, milk, and in these conditions (Bengoumi and Faye 2002). Unlike small desert animals, camels are continuously exposed to heat, solar radiation, and lack of water and food. The camel's exceptional performance in the hot desert climate results from unique behavioral, anatomical, and physiological features to economize water by reducing the metabolic rate and regulating body temperature (Tb). The so‐called “adaptive heterothermy” in the dromedary camel was discovered more than 50 years ago (Schmidt‐Nielsen et al. 1957). In the absence of heat stress and with free access to water, the daily rhythm (controlled by circadian processes; see (El Allali et al. 2013) in rectal temperature presents an amplitude of about 2°C (the animal being then a perfect thermoregulator or homeotherm). During dehydration and heat stress, the amplitude of daily Tb rhythm exceeds 6°C (thermoconformer or poikilotherm) (Schmidt‐Nielsen et al. 1957). This variation in Tb in great excess of the limits of homeothermy has been advanced as a key adaptation of camels to their arid life (Schmidt‐Nielsen 1964), (Schmidt‐Nielsen et al. 1967), (MacFarlane et al. 1963), (Dahlborn et al. 1992), (Schroter et al. 1989), (Zine‐Filali 1991), (Ayoub and Saleh 1998), and (Bengoumi and Faye 2002). High amplitude in daily Tb rhythm is thus induced in the camel by privation of water and exposure to high Ta. Under these conditions, the Tb rhythm seems partly to follow that of Ta.

Our aim was thus to characterize the daily/circadian pattern of the Tb rhythm, in the specific conditions of adaptive heterothermy in response to daily heat with or without additional dehydration. Even if the capacity of the camel to exhibit a heterothermic state has been confirmed by different independent groups (Schroter et al. 1989), (Bengoumi and Faye 2002), (Robertshaw and Zine‐Filali 1995) and (Ayoub and Saleh 1998), one author (Al‐Haidary 2005) challenged that observation. Consequently, using the new technical tools developed to follow circadian parameters of Tb (see El Allali et al. 2013), we have characterized the adaptive heterothermic process according to the circadian/daily expression of Tb. It is also known that during experimental dehydration and heat stress, camels greatly decrease their food consumption. To our knowledge, the consequence of this decrease in food intake on the adaptive heterothermic process has not been previously studied. We thus decided to investigate the possible effects of food shortage on this process.

## Experimental procedures

### Animals

All the experimental procedures reported in this study were carried out in accordance with the recommendations for animal farm husbandry, experimentation, and surgery from the Hassan II Agronomy and Veterinary Institute of Rabat and of the Moroccan Ministry of Agriculture.

Six animals used were adult female camels (9–13 years), healthy, and nonpregnant of the local Moroccan variety. The experiments took place between July and August 2010, and during July 2011, in the veterinarian institute in Rabat, Morocco (34°1′12″N/6°49′48″W). The animals were kept for both experiments in controlled sheltered stables designed to monitor photoperiod and Ta. The animal could stand or sit in sternal recumbency comfortably (sitting up on the brisket with the legs tucked under the body, the natural sitting position in camel). LD cycle 12:12 was maintained automatically with timers, light on at 8:00 am and off at 08:00 pm (about 350 lux during the lighting period). Entrance of stable was gated with Safety Access System (SAS) to avoid external light exposure. “Cold” ambient temperature of 20–25°C (at the end of dark period) was obtained using six air conditioners with total power of 75,000 BTU. High ambient temperature (38–46°C at the end of light period) was obtained using nine electric heaters. The relative humidity (RH) of ambient air was, on average, 30% as measured by a hygrometer (Model STHW, Thermo Hygrometer electronic).

### Surgery and Tb measurement

Tb was recorded using small temperature‐sensitive data loggers (Thermochron^®^: iButton, DS1922L, Dallas Maxim Integrated Products, Dallas, TX) embedded in a Paraffin/Elvax coating (Mini Mitter, Sunriver, OR) to make them waterproof and biologically inert. After waxing, the logger weighed 3.62 g and had a maximum diameter and a maximum height of 18.76 mm and 8.74 mm successively. IButtons had been programmed to record every 10 min on 16‐bit resolution (0.0625°C). The operating temperature range of Thermochron iButtons used was −40°C to +85°C (for further details on the use of iButtons, see (Davidson et al. 2003). Eight days prior to the study initiation, all camels were implanted surgically with such data loggers. The procedure was performed in fasting animals, food being withheld for 24 h and water for 12 h before surgery. Camels were tranquilized intravenously with 0.05 mg/kg of xylasine (Rompun; Bayer, Leverkusen, Germany). The camels were then maintained in sternal recumbency and the left flank was shaved and the surgical site (implantation site validated in former experiment, (El Allali et al. 2013) sterilized with 1% iodine solution and rinsed with 70% alcohol. For analgesia, 2% lidocaine (xylocaine Laprophan S.A., Maroc) at a dose of 10 mg/kg was infiltrated at the site of operation. The loggers were implanted under the deep musculature of the left flank (between the internal oblique muscle and transversus abdominis muscle), via a vertical incision (6 cm length) in the paralumbar fossa. Incisions were then sutured and treated with a topical antiseptic spray (Aluspary, Vetoquinol, Lure, France). Each animal was given postoperative therapy, a long‐acting antibiotic (100 mg IM, oxytetracyclin, ENGEMYCIN 10%, Intervet/Schering‐Plough Animal Health, Milton Keynes, UK). At the end of experiment, the data loggers were surgically removed using the same protocol.

The implantation site was validated in earlier experiments performed in our laboratory. These involved implanting iButtons in different places within the camel's body: in the right and left flank, in the thigh between muscles, intrahump, intravaginal; and to control the exchange with the ambient temperature, subcutaneously at the level of the rib. Rectal temperature (known to be reliable and close to the internal temperature) was also measured in camels. Our results (partly reported in ref El Allali et al. (2013)) show that the body temperature recorded at the level of the flank is very close to the rectal temperature.

Ta was assessed continuously by an iButton placed in the center of a sheltered room. Data were read out by the software OneWireViewer (Maxim/Dallas, Dallas, TX) and were processed by Excel (Microsoft Office, Microsoft, Issy‐les‐Moulineaus, France) and Sigma Plot^®^ (SPSS ASC GmbH, Erkrath, Germany).

### Experimental design

#### The study consisted of two experiments

Experiment 1 was designed to define the process of heterothermy in dehydrated camels under heat stress conditions. It was carried out over three successive periods, namely, a hydration period of 8 days (high amplitude of Ta cycle, normal hydration), a dehydration period (water deprivation) of 19 days, and finally a rehydration period of 2 days.

Experiment 2 was designed to investigate the effect of the reduction of food intake observed during dehydration (i.e., dehydration‐induced food intake decrease) in experiment 1 on the circadian Tb rhythm of camels kept in the same conditions of Ta and photoperiod, but with full access to water. This experiment was also conducted over three successive periods, namely, a control period (8 days), a decreased food period (19 days), and finally a refeeding period (2 days).

Water intake was measured for each camel during hydration and rehydration period (experiment 1) (see Tables 1 and 2). To keep the water fresh throughout the day, we chose to change it twice daily at 09:00 and at 17:00, while food was provided once a day at 09:00. During experiment 1 and two periods of experiment 2 (control and refeeding periods), each camel received 2 kg of a specific camel compound feed (Maraa^®^: Alf Sahel: Maraa^®^ contains: 1 – Cereals: Corn, barley; 2 – Wheat bran, wheat fiber; 3 – Oilcake: soybean, sunflower; 4 – Compound vitamin mineral. Guarantees percent: Minimum: 5 – Crude protein content: 14%; 6 – Fat: 2%; 7 – Phosphorus: 0.5%; 8 – Calcium: 0.8%. Maximum: 9 – Minerals: 10%; 10 – Crude cellulose: 14%; 11 – Vitamins UI/100 kg; 12 – vitamin A: 500,000; 13 – vitamin D: 150,000; 14 – vitamin E: 5000, humidity: 13%) and 3 kg of wheat straw. During the decreased food period of experiment 2, we supplied the same food but with a progressive decrease of the quantity mimicking the spontaneous decrease in food intake observed in experiment 1. On the last day of this period, we achieved a loss of 35% for compound food and 76.6% for wheat straw.

**Table 1.** Water intake (liters) during last 4 days of hydration period (experiment 1).

Camels	Water intake (liters)			
	Day 5	Day 6	Day 7	Day 8
B	10	10	11	9
C	8	6	9	7
E	10	10	11	10
F	8	5	5	9
G	10	16	10	8.5
H	10	10	15	8

**Table 2.** Water intake (liters) during rehydration period (experiment 1).

Camels	Day 28		Day 29	
	Water intake (liter)	Time (min)	Water intake (liter)	Time (min)
B	53	7	30	1440
C	56	10	10	1440
E	53	10	32	1440
F	61	5	29	1440
G	55	2	27	1440
H	56	10	30	1440

### Control of dehydration

To assess the degree of dehydration, we monitored the evolution of several biological parameters. Blood samples were collected from one of the jugular veins in vacuum tubes with anticoagulant (heparin) before feeding during hydration, dehydration, during rehydration periods of experiment 1, and during all periods of experiment 2. Hematocrit (Ht) and hemoglobin (Hb) concentrations were determined immediately after sample collection. Ht was determined using hematocrit tubes. Using the Colorimetric Method Manual, we measured the Hb concentration (RANDOX Laboratories, Antrim, UK). Plasma was then centrifuged at 3500 g for 10 min, and the total plasma protein concentration (PT) was measured (g/100 mL) of each sample, in duplicate, on a refractometer (ATAGO SPR‐T2 Protein 0–12^9/100 mL^).

Changes in body weight were measured to evaluate the degree of dehydration and associated decrease in food intake in experiment 1, and to determine the effect of food shortage on body weight during experiment 2. The estimation of body weight was performed using the barymetric formula of (Boué 1949) adapted after by (Graber 1966):
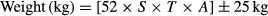
where *S* stands for shoulder height (in meter), *T* is the thoracic girth (in meter), and *A* is the abdominal girth (in meter).

We calculated the loss of body weight in each animal and in both experiments using the following formula:
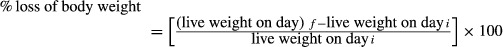
where day *i* is the day on which one needs to calculate the loss of body weight and day *f* is the last day of hydration period (experiment 1) or the last day of control period (experiment 2).

The healthy state of the camels was monitored every day by a clinical examination. The respiratory and heart rates were specifically assessed daily at 08:00 (data not shown).

### Statistical analysis

Tb data obtained from all loggers provided continuous measurements for 29 days from each animal and for each experiment. The data were consolidated by pooling the 6 × 10 min interval data points obtained from each logger for each hour of measurement to produce 24 average hourly temperatures for each animal for each day. These hourly averages for the six animals were, in turn, averaged to produce a mean hourly temperature for them as a group. These averages were further pooled for all study days or for the component days of the study period to provide a comprehensive overview of Tb throughout the recording period. For example, in experiment 1, the data were averaged in each 24‐h interval and then averaged over the last 4 days for both periods: hydration and dehydration and during the first day for rehydration period. The amplitude was computed as the difference between the highest and the lowest daily measurements, and averaged as for mean.

To assess the influence of the light–dark cycle on Tb, the phase relationships between the dark–light transition and the peak body temperature were calculated and followed during the different phases of the experiment (experiment 1 and experiment 2).

To assess the influence of the environmental Ta on Tb during experiment 1, we studied the relationship between Ta and Tb per 24 h over 10‐min intervals of both variables using the slope of identity line “Ta = Tb”. When the abscissa and ordinate are on the same scale, the identity line forms a 45° angle with the abscissa. We made a translation of this line to be tangential to the curve Tb = *f* (Ta). This tangent is presented as a dotted line in Fig. 4.

Means ± standard error of the mean (SEM) are reported. One‐way repeated measures analysis of variance (ANOVA) followed by Tukey's post‐hoc test was applied to determine the significant effect of different experimental conditions on the parameters studied. We assumed statistical significance at *P* value <0.05. The data were analyzed using the software STATISTICA 10.0 (Stat Soft Inc, Maison Alfort, France).

## Results

### Effects on rhythm of Tb

**Figure 1.**
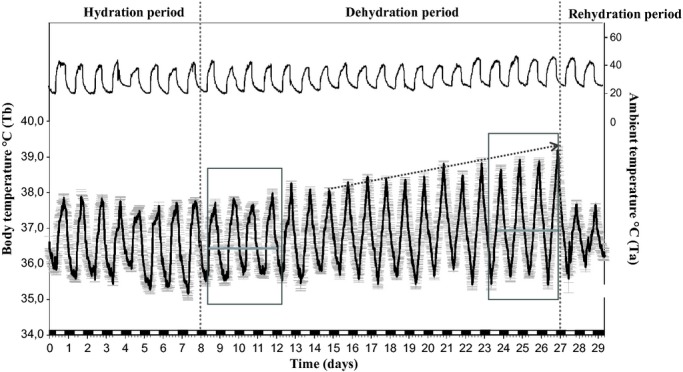
Average rhythm of Tb of six camels in Experiment 1. Each data point corresponds to the mean (±SEM) of the six camels. Data were collected every 10 min for 29 consecutive days Tb rhythm (Black line down). Ta is represented by the upper curve. The vertical dotted lines separate the different periods of the experiment. The arrow shows the progressive increase of the amplitude of Tb rhythm after 7 days of water deprivation. Horizontal gray lines indicate the mean of Tb during the first (36.39°C ± 0.04) and last 4 days of dehydration period (36.95°C ± 0.05). The white and black blocks at the bottom of the figures denote the duration of the light and dark phases of the light–dark cycle, respectively.

**Figure 2.**
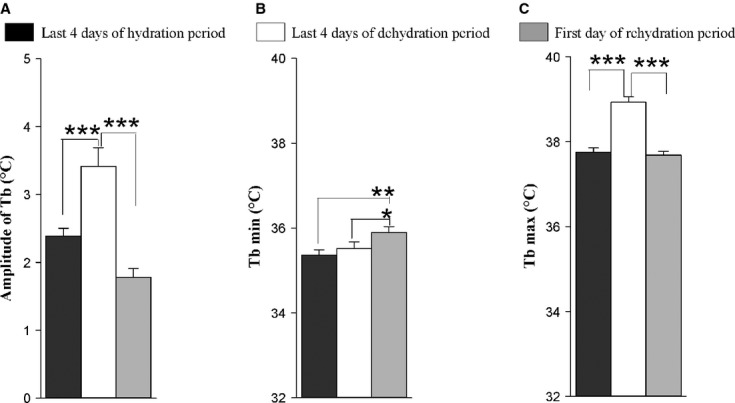
(A) Amplitudes of average (±SEM) Tb of six camels. (B) Average (±SEM) of minimum of Tb rhythm of six camels. (C) Average (±SEM) of maximum of Tb rhythm of six camels. Data were computed over the last 4 days of each period of hydration (black bars) and dehydration (white bars) and the first day of rehydration period (gray bars). *0.01 < *P* values≤0.05, **0.001 < *P* values≤0.01, ***P values≤0.001.

The average daily Tb rhythm for the six camels maintained under a 12L/12D cycle and under Ta cycles with 38–46°C in the day and 20–25°C at night (Experiment 1) is shown in Fig. 1. The Tb show robust daily rhythmicity. The lowest Tb was observed in the morning, and the highest in the late afternoon. During the hydration period, the amplitude of average Tb rhythm was 2.39 ± 0.11°C (Fig. 2A) with a minimum of 35.37 ± 0.13°C (Fig. 2B) noted in the morning and a maximum of 37.76 ± 0.10°C recorded in the evening (Fig. 2C). The amplitude began to rise gradually from the 7th day of water deprivation (Day 15). After 19 days of dehydration (Day 27), the amplitude of the average Tb rhythm reached a value of 3.8 ± 0.25°C (Fig 2A), with a minimum of 35.42 ± 0.17°C (Fig. 2B) noted in the morning and a maximum of 39.22 ± 0.11°C (Fig. 2C) recorded in the evening. The amplitudes of the daily Tb rhythm calculated over the last four successive days of dehydration period were between 3.0 to 4.47°C in five of six camels (Fig. 2A).

**Figure 3.**
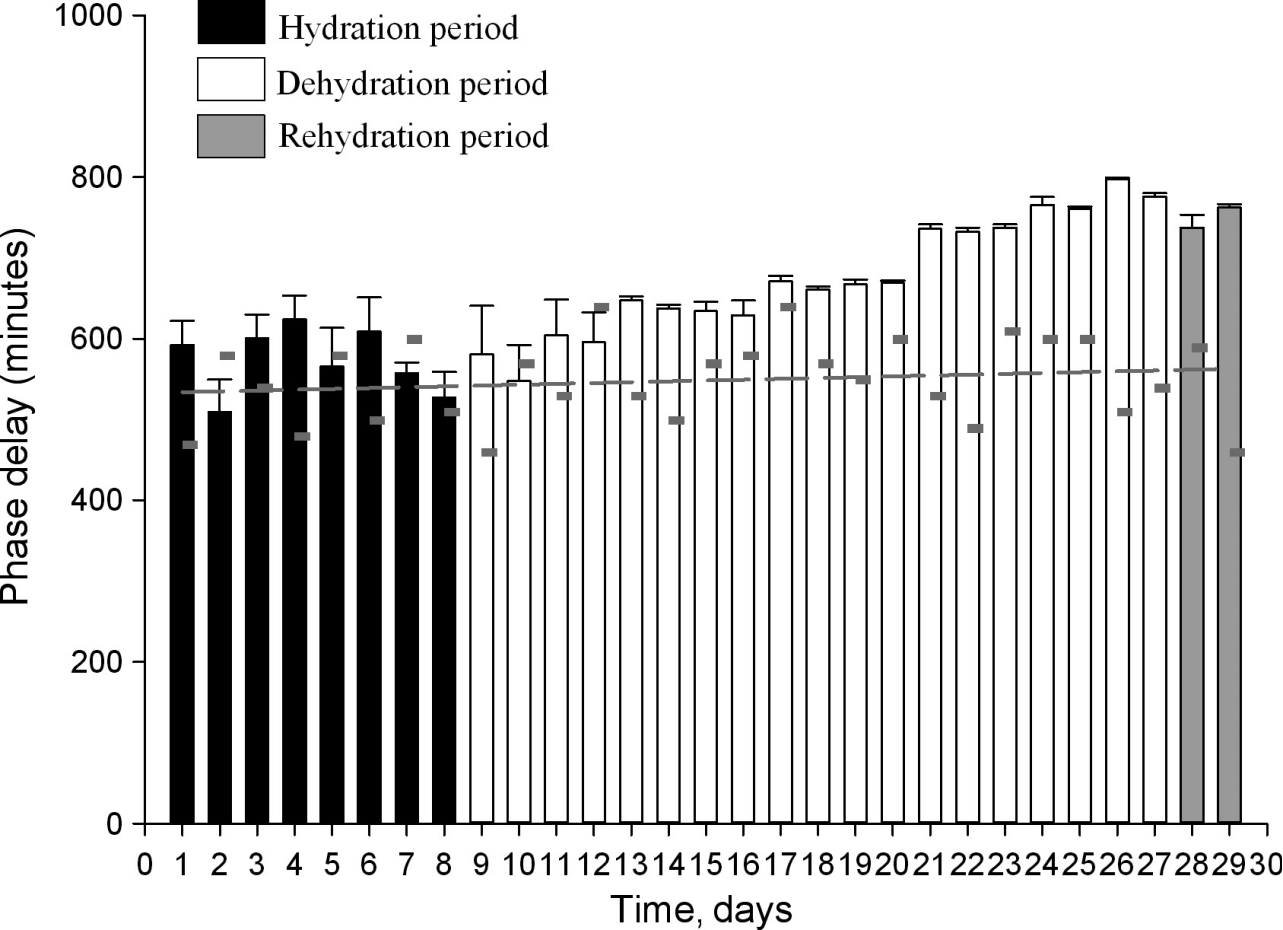
Phase relationship (±SEM) between the maximal Tb rhythm and the time of the light–dark transition. Data were computed over 29 days on experiment 1: during 8 days of hydration period (black bars), 19 days of dehydration period (white bars), and during 2 days of rehydration period (gray bars). Small gray dashes indicate the moment of maximal Ta and dark gray line indicates nonlinear regression function: *f* = *y*0 + *a* * *x*.

In addition to an effect on amplitude, we also observed a change in phase relationship with the light–dark cycle. In camel H, the maximum of the Tb rhythm was observed at 589 min (9 h 48 min) after the dark–light transition on the last day of the hydration period, and at 779 min (12 h 58 min) on the last day of the dehydration period representing a phase delay of more than 3 h (Table 3). This observation was confirmed by statistical analysis of the six camels (527.33 min ± 31.77 versus 775.67 ± 4.22) (Table 3). Interestingly, this clear delay in the maximal Tb rhythm with respect to the light–dark cycle was progressive during the dehydration period (Fig. 3). Due to the technical constraints in the animal facilities, the exact moment of maximal temperature varied each day but linear regression demonstrated that globally the maximum was constant, and that variability in the Ta maximum was not responsible for the observed progressive phase delay in Tb. With regard to the amplitude, the Tb rhythm showed an immediate response to rehydration, decreasing from 3.8 ± 0.25°C (Day 27) to 1.78 ± 0.13°C (Day 28) (Fig. 2A). A repeated measures one‐way ANOVA revealed a statistically significant difference in the mean values of amplitude of the Tb rhythm among the three periods of experiment 1 (*F*_(2,10)__=_ 41.391, *P* value = 0.00001). The increase in amplitude of Tb was significant between the dehydration period and each of other two periods (*P* value <0.05) (Fig. 2A).

**Table 3.**
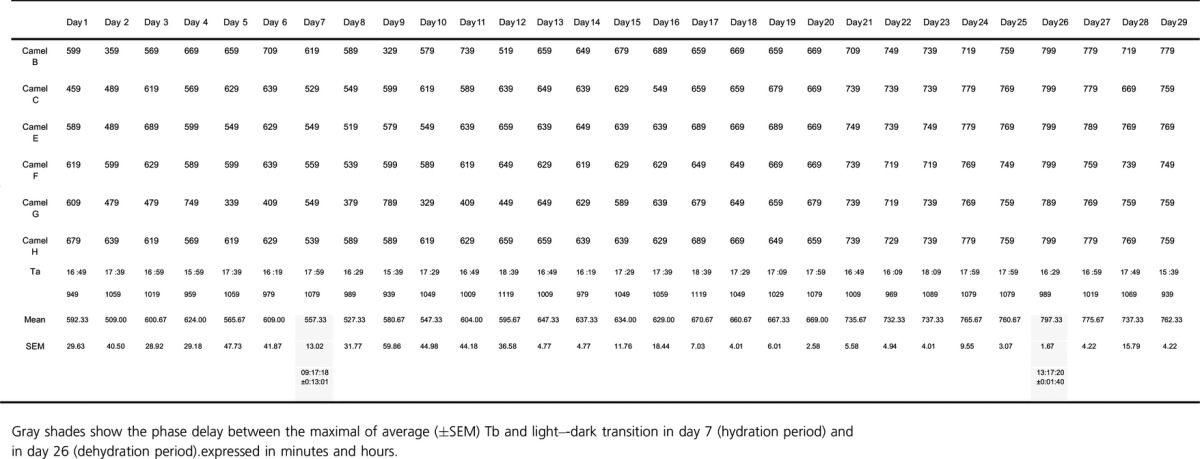
Phase delay (minutes) between the maximum of Tb and the dark–light transition through the days of experiment 1.

To show unambiguously the progressive increase of the Tb rhythm amplitude during the dehydration period, we compared the Tb rhythm of all animals during the first 4 days versus the last 4 days in this period (Fig. 1). In the first 4 days of the dehydration period, the mean Tb_min_ of the six camels was 35.52 ± 0.15°C, and the same value (35.52 ± 0.16°C) was observed during the last 4 days of this period (Fig. 2B). By comparing the Tb_max_ during the first 4 days of the dehydration period versus the last 4 days of the same period, it can be seen that the increase in amplitude from the 7th day of dehydration occurs by an increase in Tb_max_ and not by a decrease in Tb_min_. (Fig. 2B and C).

**Figure 4.**
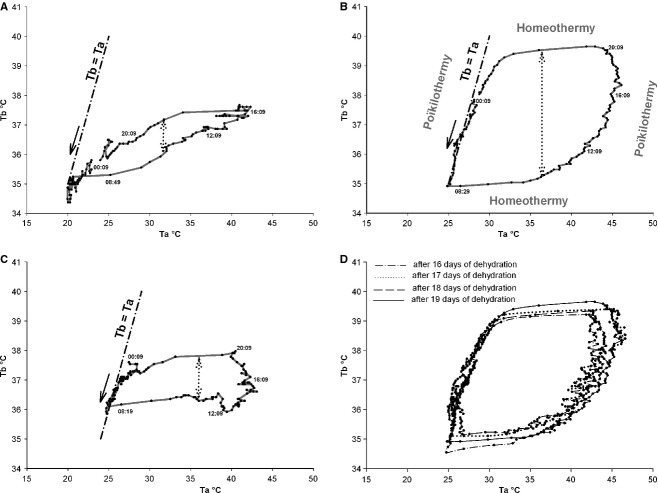
Records of 24 h of Tb (solid line) from a camel H as function of Ta, during the last day of hydration period (A), the last day of dehydration period (B), throughout the first day of rehydration period (C), and over the last four consecutive days of dehydration period (D). Data were collected every 10 min. Dash‐dotted lines indicate the slope of identity line “Tb°C = Ta°C”. The dotted arrows show schematically the amplitude of the Tb rhythm. The direction of the black arrows indicates the course of time.

To have a clear representation of the effect of Ta on the thermoregulation system in camels, we also studied the evolution of Tb rhythms as a function of the Ta for each day during the hydration, dehydration, and rehydration periods. To facilitate the presentation of results, we chose to represent the daily Tb rhythm of a representative camel as a function of the Ta during the last day of the hydration and dehydration periods, and during the first day of the rehydration period (Fig. 4A, B and C). This analysis confirmed that throughout the hydration period the camel's Tb varies very little, the amplitude of Tb rhythm of the representative camel (H) in the last day of hydration period being only 2.6°C (Fig. 4A). During the dehydration period, the variation between morning and evening Tb increases progressively with the degree of dehydration. At the 19th day of water deprivation, the Tb of the representative camel (H) varied by 4.73°C (Fig. 4B). After a severe dehydration period, the immediate effect of the availability of water in reducing the daily Tb fluctuations is obvious: on the first day of the rehydration period, the amplitude of Tb rhythm of the representative camel (H) was 2.4°C (Fig. 4C). Similar data were obtained for the six camels (data not shown). This thorough analysis of the rhythm of Tb as a function of Ta revealed the existence in the camel of a daily heterothermic variation of Tb during the dehydration period. As shown in the representative camel (H), during night and early morning, the curve of the dehydrated camel's Tb is tangential to the slope of the identity line (dotted line Fig. 4B). Camel's Tb passively follows the Ta, that is, the camel behaves as a thermoconformer (poikilotherm). During the morning Ta tends to increase, so the camel regulates its Tb to remain stable, probably by peripheral vasodilatation, that is, it becomes a thermoregulator (homeotherm). Thereafter, throughout the afternoon, the Ta continues to rise but the camel stops regulating Tb. The animal accumulates heat during the day, with a consequent rise in Tb. The camel once again enters a state of thermoconformer. This stored heat is then dissipated nonevaporatively at night when the heat load is lower (Schmidt‐Nielsen et al. 1957). Subsequently, the camel will once again regulate Tb to keep it stable (homeothermic). These results clearly demonstrate, depending on water availability, the presence of strong plasticity in the thermoregulatory system with a daily double switch between homeothermic and poikilothermic states. Interestingly, even during dehydration, the relationships with the light–dark cycle are maintained, as represented in Fig. 4D. On the last four consecutive days of dehydration period (in camel H for example), not only the amplitude but the timing of this heterothermic variation is the same.

**Figure 5.**
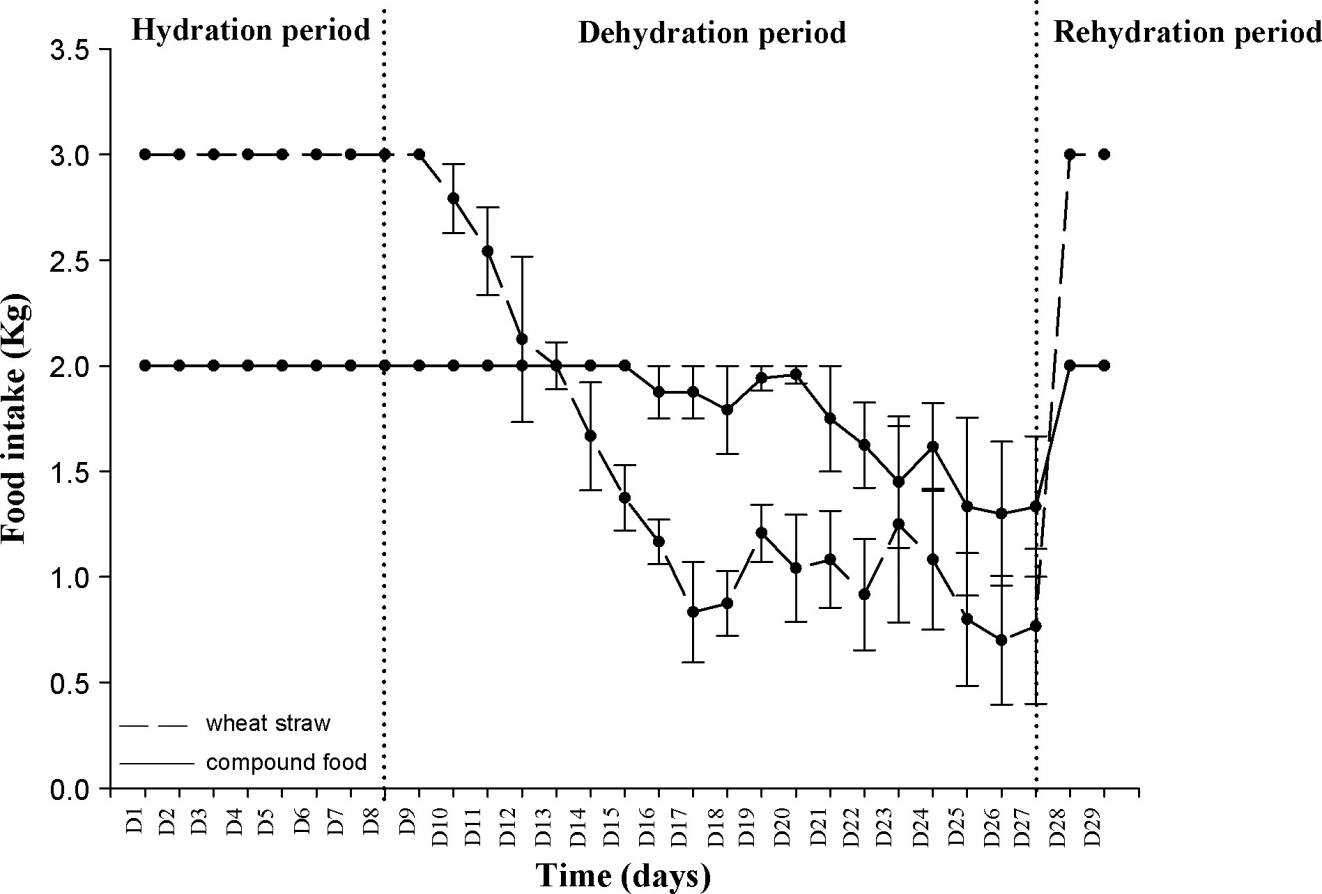
Twenty‐four‐hour means (±SEM) of food intake of compound food (open circle) and of wheat straw (dark circle) of six camels during experiment 1. The vertical dotted lines separate the different periods of the experiment. During dehydration period, a fall of food intake of 35% for the compound food and of 76.6% for the wheat straw were noted.

Next, we studied in detail the dehydration‐induced decrease of food intake in dehydrated camels submitted to a heat stress. During the initial period of hydration, camels consumed the entire food ration distributed (2 kg of compound food and 3 kg of wheat straw). After 7 days of water restriction, food intake of compound food dropped progressively. This decrease became greater from the 12th day of dehydration until the last day of this period. For wheat straw, we observed an immediate (second day) decrease in food intake during the dehydration period (Fig. 5). We noted that there was a greater decrease in food intake for straw than for compound food. Therefore, after 19 days of water deprivation camels reduced their food intake of compound food by 35% but by 76.6% for straw (Fig. 5). Rehydration led to a rapid and immediate recovery of appetite for straw and compound food. Body weight dropped progressively until the day of rehydration and after 19 days of dehydration the camels had lost 23.43% of their initial weight (Table 4). Partial recovery of initial body weight was observed on the first day of the rehydration period. One week was needed to obtain a full recovery (data not shown). One‐way repeated measures ANOVA showed a significant difference in the mean values of body weight among the three periods of experiment 1 (*F*_(2,10)__=_ 65.877, *P* value = 0.000002). A Tukey's post‐hoc test indicated that the decrease of body weight was significant between the dehydration period and each of other periods (*P* value<0.05).

**Table 4.** Effect of water deprivation on camel body weight (Experiment 1).

Camels	% loss of body weight
Camel B	22.87
Camel C	19.94
Camel E	25.1
Camel F	22.3
Camel G	22.3
Camel H	28.0
Mean	23.43

**Figure 6.**
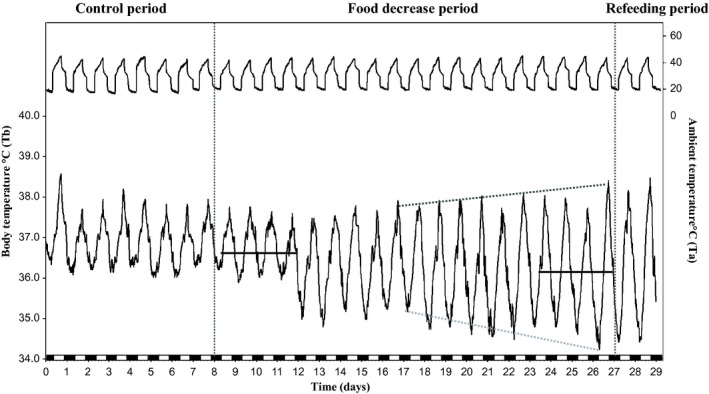
Records of Tb from a camel (H) in experiment 2 (black line down). Data were collected every 10 min for 29 consecutive days. Ta is represented by the upper curve. The white and black blocks at the bottom of the figure denote the duration of the light and dark phases of the light–dark cycle, respectively. Dark gray and gray dotted lines represent the evolution of maximum en minimum of the Tb rhythm. Horizontal black lines indicate the mean of Tb during the first (36.74°C ± 0.02) and last 4 days of food decease period (36.16°C ± 0.04).

**Figure 7.**
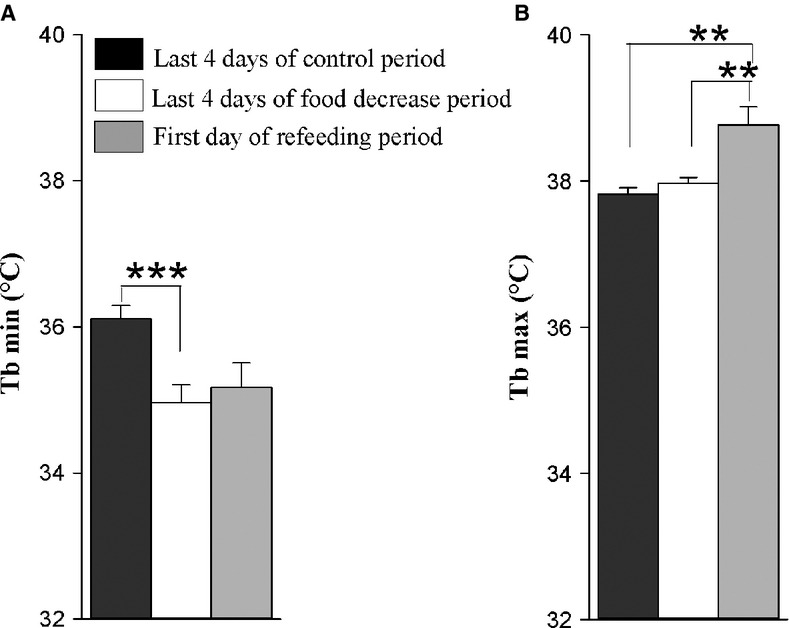
Average (±SEM) minimum (A) and maximum (B) of Tb rhythm of six camels. Data were computed over the last 4 days of each period of control (black bars) and food decrease (white bars) and the first day of refeeding period (gray bars). **0.001 < *P* values≤0.01,***P values≤0.001.

In Experiment 2, we analyzed Tb rhythms in pair‐fed camels exposed to heat stress with free access to water to evaluate a possible role of decreased food intake in the establishment of a heterothermy strategy in the camel. The recorded Tb of a representative food‐restricted camel (H) is shown in Fig. 6. Under food decrease conditions, a daily rhythm of Tb was still observed. The Tb_min_ during the first 4 days of the food decrease period was 36.08 ± 0.04°C; it dropped to 34.97 ± 0.24°C over the last 4 days of the same period (Fig. 7A). In the first 4 days of the food decrease period, the mean of Tb_max_ of our six camels was 37.77 ± 0.07°C. A close value of 37.93 ± 0.06°C was noted during the last 4 days of this period (Fig. 7B).

**Figure 8.**
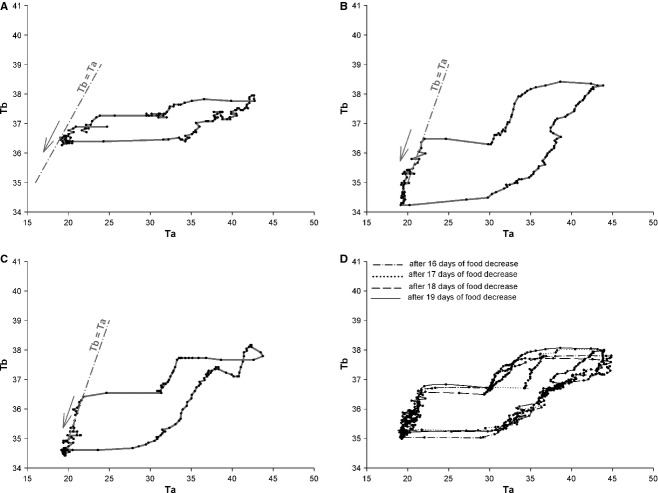
Records of 24 h of Tb (solid line) from a camel H as function of Ta, during the last day of control period (A), the last day of food decrease period (B), throughout the first day of refeeding period (C), and over the last four consecutive days of food decrease period (D). Data were collected every 10 min. Dash‐dotted lines indicate the slope of identity line “Tb°C = Ta°C”. The direction of the arrows indicates the course of time.

We also studied, in a representative camel (H), the evolution of the Tb rhythm as a function of Ta for each day during the three periods. This analysis (Fig. 8) revealed that after food restriction as after dehydration, there was strong daily plasticity in the thermoregulatory system with a daily double switch between homeothermic and poikilothermic states.

**Figure 9.**
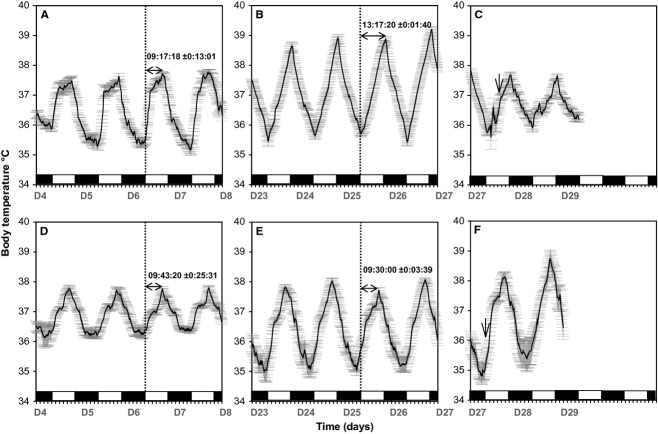
Means (±SEM) of Tb rhythm of six camels in both experiments 1 and 2. (A) during the last 4 days of hydration period, (B) during the last 4 days dehydration period, and (C) during 2 days of rehydration period in experiment 1. (D) during the last 4 days of control period, (E) during the last 4 days progressive decrease in food period, and (F) 2 days of refeeding period in experiment 2. The vertical dotted lines indicate the time of light on (08:00 h) for days 7 and 26 in both experiments 1 and 2. The horizontal arrows show the phase relationship between the maximal of Tb and light–dark transition. The vertical arrows illustrate the beginning of the rehydration (C) and of refeeding (F). The white and black blocks at the bottom of the figure denote the duration of the light and dark phases of the light–dark cycle, respectively.

Combining experiments 1 and 2, we compared the average Tb of six camels during the last 4 days of the hydration and dehydration periods versus the last 4 days of the control and food decrease periods, and during the first day of the rehydration period versus the first day of the refeeding period (Fig. 9). A slight variation was noted in mean Tb of camels kept under hydration periods during experiments 1 and 2 (Fig. 9A and D. This difference may correspond simply to a change in pattern of the Ta cycle. Indeed, depending on the outside environmental temperature, its requires different times to heat up or cool down the shelder; The repeated measures one‐way ANOVA revealed an overall significant effect of the three periods of both experiments 1 and 2 on the mean Tb of the six camels (*F*_(2,10)__=_ 14.5, *P* value = 0.00110 and *F*_(2,10)__=_ 8.588, *P* value = 0.00675, respectively). Tukey's post‐hoc pair‐wise comparisons showed, on one hand, the increase in mean Tb was significant between the dehydration period and each of the other two periods in experiment 1 (*P* value < 0.05), and on the other hand, the decrease in mean Tb was significant between the food decrease period and control period in experiment 2 (*P* value < 0.05).

**Figure 10.**
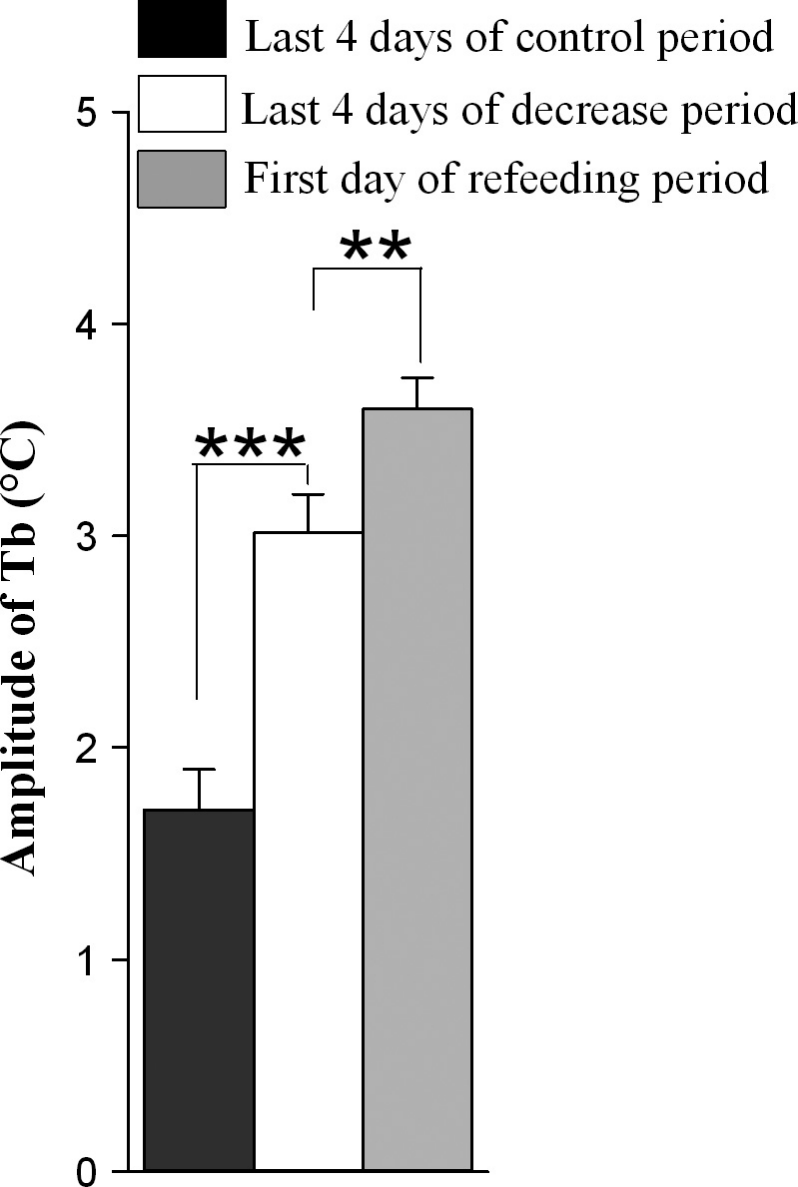
Amplitudes of average (±SEM) Tb of six camels. Data were computed over the last 4 days of each period of control (black bars) and food decrease (white bars) and the first day of refeeding period (gray bars). **0.001 < *P* values≤ 0.01; ***P values≤0.001.

In both experiments 1 and 2, we found high amplitudes of Tb over the last 4 days of the dehydration and food decrease periods. They were 3.41 ± 0.28°C (Fig. 2A) and 3.01 ± 0.18°C (Fig. 10), respectively. The increase in amplitude over the last day of food decrease period was due to a decrease in Tb_min_ (Fig. 7A) and not due to an increase in Tb_max_ (Fig. 7B) as in experiment 1. It is also clear from these experiments that the phase delay of the maximum of the Tb rhythm with respect to the light–dark transition, as observed during the dehydration period, is not observed in the food‐restricted animals (Fig. 9).

On the first day of the rehydration and refeeding periods, amplitudes of Tb were 1.78 ± 0.13°C (Fig. 2A) and 3.59 ± 0.15°C (Fig. 10), respectively. Whereas availability of water has an immediate effect in reducing the amplitude of Tb rhythms, refeeding has a late effect on this parameter. Tb went back to control values within a few days (data not shown).

In experiment 2, after 19 days of a progressive food intake decrease, the camels lost only 12.99% body weight (Table 5). After 2 days of refeeding with 2 kg compound food and 3 kg of wheat straw, partial recovery was observed. The total recovery to control levels was seen after 1 week (data not presented). Also, using one‐way repeated measures ANOVA, a significant difference was found in the mean values of body weight among the three periods of experiment 2 (*F*_(2,10)__=_ 35.336, *P* value = 0.00003). A post‐hoc test showed that the decrease in body weight was significant between the food decrease period and control period (*P* value < 0.05).

**Table 5.** Effect of progressive food decrease on camel body weight (Experiment 2).

Camels	% loss of body weight
Camel B	9.66
Camel C	9.87
Camel E	14.34
Camel F	19.06
Camel G	14.05
Camel H	10.94
Mean	12.99

### Effects on hematological parameters

Values of total protein (PT) and hemoglobin (Hb) increased progressively during the dehydration period, from 7.2 to 8.3 g/100 mL and from 10.32 to 16.09 g/dL, respectively. They reached a maximum after 15 min of rehydration: 8.9 g/100 mL and 16.60 g/dL, respectively. After 2 h of rehydration, PT and Hb were decreased. Based on the results of the one‐way repeated measures ANOVA, dehydration conditions in this experiment significantly influenced these parameters (PT: *F*_(12,60)_ = 51.088, *P* value = 0.00000, Hb: *F*_(12,60)_ = 8.2076, *P* value = 0.00000). Hematocrit (Ht) was unchanged during the dehydration period, thus confirming previous observations in the same species (Kamili et al. 2013). No change was observed in the PT, Ht, and Hb throughout experiment 2, suggesting that the volume of plasma was not markedly modified.

## Discussion

**Figure 11.**
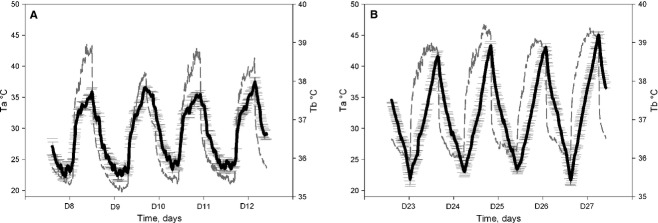
Superposition of average of Tb rhythm (±SEM) (black line) calculated in our six camels, during the first 4 days (A) and last 4 days (B) of dehydration period with the daily rhythm of Ta (gray line).

Our observations clearly establish that water deprivation during exposure to daily heat has a significant effect on the amplitude of daily/circadian Tb rhythms in the camel (adaptive heterothermy). This confirms a number of previous observations (Schmidt‐Nielsen et al. 1957), (Schmidt‐Nielsen et al. 1967), (Dahlborn et al. 1992), (Zine‐Filali 1991), (Bengoumi and Faye 2002), (Bekele et al. 2013) and is in contrast to the findings of (Al‐Haidary 2005). The use of small temperature‐sensitive data loggers continuously recording Tb without any manipulation of the animals, as well as the use of rooms with controlled conditions, has permitted long‐term detailed study in adaptive heterothermy and the description of novel aspects of its regulation. When animals are challenged with both dehydration and heat stress, we observed for the first time that adaptive heterothermy corresponds to a daily alternation of homeothermic and poikilothermic periods, characterized by a double switch. This strong plasticity in the thermoregulatory system corresponds to a daily cycle of Tb entrained by the light–dark cycle (see Fig. 4D). As we have already demonstrated that the daily Tb rhythm in the camel is dependent on the master circadian clock (El Allali et al. 2013), it is likely that this daily plasticity in the thermoregulatory system when animals are water deprived and submitted to heat stress also represents a circadian process. This interpretation is hypothetical, and its validation will require extended studies in camels kept under constant conditions (constant dark or constant light). The clear relationships between the phase delay of the peak in Tb rhythm (with respect to the dark–light transition) and duration of dehydration (a phenomenon not observed when animals are only food deprived: Fig. 9), speak in favor of the circadian nature of the adaptive heterothermy process, dehydration changing either the functioning of the master clock or the synchronizing effect of the light–dark cycle on the clock. However, it is not possible to provide a definitive conclusion at the present time. Indeed, as clearly demonstrated in the present work, Tb rhythm in dehydrated camels is characterized by a direct dependence of the ambient temperature (poikilothermy), at least during two periods of the day–night cycle. Thus, we cannot totally exclude that the measured phase delay associated with dehydration corresponds simply to a change in the pattern of the Ta cycle. The superimposition of the averaged Tb rhythms of the six camels, during the first 4 days and last 4 days of dehydration period, with the daily rhythm of Ta seems to support that suggestion (Fig. 11). Independently of the mechanism involved, the phase change of the Tb rhythm peak will induce drastic changes in the physiology of the animals. Indeed, optimal and anticipatory temporal organization of biological functions in relation to periodic changes of the environment relies on a complex network comprising a master circadian clock (SCN), synchronizing inputs, various outputs as well as multiple central and peripheral oscillators (Pevet and Challet 2011), (Davidson et al. 2003; Dibner et al. 2010). It is the complex interactions of multiple outputs from the SCN that drive the circadian expression of events within the body. Tb rhythm is an output of the SCN which is known to be one of the potent cues used for resetting peripheral oscillators, in a global entraining temporal organization of functions (Brown et al. 2002, 2002; Buhr et al. 2010). In the camel, dehydration‐induced changes in the phase relationships between the light–dark cycle and the Tb rhythm will thus consequently induce a state of internal desynchronization between various functions. This response may be a part of the adaptive mechanism to desert environment.

In the camel, it has also been demonstrated that the daily Ta cycle is able to entrain the master circadian clock, thus highlighting that Ta is a true zeitgeber (El Allali et al. 2013). The protocol validated in this study will also permit to test in these specific conditions, if the zeitgeber effect of Ta cycle with higher amplitude is able, as suggested by (El Allali et al. 2013), to counteract the zeitgeber effect of the light–dark cycle, a fundamental question in mammalian circadian biology. It will also lead to determine more precisely the functioning of the master clock and the circadian network in dehydrated and heat‐stressed camels.

The use of data loggers over a long period of time has also facilitated understanding the interpretation of (Al‐Haidary 2005). In the present work, we observed that the increase in amplitude of Tb starts after 7 days under heat stress and water deprivation. In the work of (Al‐Haidary 2005), the analysis was only done after 4 days and the protocol used by this group would have overlooked any heterothermy process.

Daily/circadian alternation of periods of poikilothermy and homeothermy appears instrumental for camel physiology. Is it also true for other mammals living in such drastic environmental conditions? Captive Idmi gazelles (*Gazella gazella*; (Al‐Johany et al. 1998), free‐living Arabian Sand gazelles (*Gazella subgutturosa marica*; (Ostrowski and Williams 2006), free‐living Arabian Oryx (*Oryx leucoryx*; (Ostrowski and Williams 2003), (Hetem et al. 2010), and Ethiopian Somali goats (Shkolnik and Choshniak 2006; Mengistu et al. 2007) are known to present such adaptive heterothermy. Recently, it has been also described that hydrated Asian elephants (*Elephas maximus*) can cope with high heat load by employing a heterothermy strategy (Weissenböck et al. 2012). This phenomenon of adaptive heterothermy seems to be a phenomenon specific to desert or semidesert animals. If we realize that human individuals are also living in such zones and have to adapt to these strong environmental conditions, it seems important to understand the physiological mechanisms involved in the development of such adaptive process, especially for its rhythmic/circadian organization viewed as a key to adapt to strong environmental changes in the desert.

In the past, it was noted that dehydration and heat stress led to decreased food consumption in the camel. To our knowledge, this observation has not yet been described in detail, and its effect on the increased amplitude of the Tb rhythm (establishment of daily adaptive heterothermy) has never been studied. In the present work, we described and quantified this phenomenon. Under water deprivation and heat stress, camels progressively reduced their food intake by 35 and 76.6% for compound food and straw, respectively. As a consequence, a concomitant decrease in body weight was noted. Rehydration led to a rapid and immediate recovery of appetite for straw and compound food. In rodents, imposed dehydration has already been shown to trigger a so‐called “dehydration‐induced food intake decrease” (e.g., (Alvarez‐Salas et al. 2012; Watts and Boyle 2010). Our question was whether this decreased food intake is an integral part of the process of adaptive heterothermy in camels, and if its relationships with the light–dark cycle can be changed. Here, we show that imposed food decrease in perfectly hydrated and heat‐stressed camels increases the amplitude of Tb rhythms. This result confirms similar findings in food‐restricted rodents (e.g., Nelson and Halberg 1986; Challet 2010). In this study, the observed decrease in food consumption due to dehydration and/or heat stress may be involved in the mechanism of adaptive heterothermy. This raises the question of the respective role of food consumption decrease and water depletion in the heterothermic response to heat stress. This question is difficult to address fully: whereas one can isolate the effect of restricted food alone, the examination of water depletion alone is virtually impossible to perform since water depletion induces the decrease in food intake in the camel. Be that as it may, comparison of the data obtained in our two experiments permits several conclusions. In hydrated camels, the increase in amplitude of Tb resulting from imposed food restriction (i.e., based on pair‐feeding according to dehydration‐induced decrease in food intake) is characterized by a strong and progressive decrease of Tb_min_ at the end of the night. This is compatible with the decrease in basal metabolism classically observed under food restriction in rodents (e.g., (McCarter and McGee 1989) and is in agreement with previous observations in the camel (Dahlborn et al. 1992; Zine‐Filali et al. 1995). Compared with the Tb values in the control period, this strong decrease in the Tb_min_ explains the lower mean of Tb, even if a very small increase of the Tb_max_ is also observed. Based on these findings, decreased metabolic rate can be interpreted as a strategy for the camel to conserve energy stores during starvation. When the camels were dehydrated (same decrease in food consumption as in food‐restricted hydrated camels), a similar large increase in amplitude of Tb rhythm was observed. In these conditions, there is a progressive and large increase in Tb_max_ at the end of the afternoon that can explain the increased mean level of Tb. Interestingly, the level of Tb_min_ seems to be only marginally affected under these conditions of heat and dehydration. Furthermore, unlike when only food intake is restricted, the decrease in minima of Tb is not observed when water is no longer available, even if the degree of food restriction (due in that case to the decrease in food intake) is similar.

Clearly, both external factors (dehydration and level of food) are involved in the process of adaptive heterothermy, probably by different mechanisms working at different levels. This view is supported by the fact that the phase shift of the maximum Tb rhythm observed in dehydrated camels is not detectable in food‐restricted animals. Based on the present findings, we can conclude that adaptive heterothermy in the Arabian camel is thus a combination of three factors interacting throughout the daily light–dark cycle: heat stress, water restriction, and the level of food intake.

## Acknowledgments

This work has been done in the framework of a PhD thesis in neurosciences performed in IAV Hassan II (Morocco) in co‐tutelle with Strasbourg University (France). We are grateful to Moroccan Ministry of Agriculture and to colleagues of physiology Unit, Parasitology Unit and all staff of Anatomy Unit from the Hassan II Agronomic Veterinary Institute in Rabat as well as from the Institute for cellular and integrative neurosciences (INCI 3212, CNRS‐Université de Strasbourg) for their help in achieving this study**.** We are also grateful to Dr A. Malan for his suggestions and help in the mathematical analysis of the data and to Dr. David Hicks for his English revision.

## Conflict of Interest

The authors declare no conflict of interest.
